# Crystal structure and Hirshfeld surface analysis of 3-octyl-4-oxo-2,6-bis­(3,4,5-tri­meth­oxy­phen­yl)piperidinium chloride

**DOI:** 10.1107/S2056989018008125

**Published:** 2018-06-08

**Authors:** Rubina Siddiqui, Urooj Iqbal, Zafar Saeed Saify, Shammim Akhter, Sammer Yousuf

**Affiliations:** a Department of Pharmaceutical Chemistry, Faculty of Pharmacy and Pharmaceutical Sciences, University of Karachi, Karachi-75270, Pakistan; b H. E. J. Research Institute of Chemistry, International Center for Chemical and Biological Sciences, University of Karachi, Karachi-75270, Pakistan

**Keywords:** crystal structure, piperidine-4-one, Mannich reaction, Hirshfeld surface analysis

## Abstract

The title compound, C_31_H_46_NO_7_^+^·Cl^−^, was synthesized by a one-pot Mannich condensation reaction. In the mol­ecule, the piperidinone ring adopts a chair conformation, and the trimeth­oxy-substituted benzene rings and octyl chain are arranged equatorially. In the crystal, centrosymmetric dimers are linked into layers parallel to (011) by N—H⋯Cl and C—H⋯Cl hydrogen bonds. A Hirshfeld surface analysis indicates that the most important contributions for the crystal packing are O⋯H (20.5%) inter­actions followed by C⋯H (7.8%), Cl⋯H (5.5%), C⋯C (1.2%), C⋯O (0.5%) and Cl⋯O (0.4%) inter­actions.

## Abstract 2

The title compound was synthesized by a one-pot Mannich condensation reaction. In the crystal, centrosymmetric dimers are linked into layers parallel to (011) by N—H⋯Cl and C—H⋯Cl hydrogen bonds. A Hirshfeld surface analysis indicates that O—H (20.5%) inter­actions make the largest contribution to the crystal packing.

## Chemical context   

Piperidine is a naturally occurring bioactive alkaloid (Hu *et al.*, 2002 ▸; Finke *et al.*, 2001 ▸; Taniguchi & Ogasawara, 2000 ▸) and the heterocyclic six-membered nitro­gen-containing piperidine ring is an essential structural part of many important drugs including paroxetine, raloxifene, haloperidol, droperidol and minoxidiln (Wagstaff *et al.*, 2002 ▸). 2,6-Diphenyl-substituted piperdine-4-one derivatives are important because of their potential biological activities such as anti­tumor, anti­microbial, analgesic, local anesthetic, anti­depressant and anti-inflammatory (Kálai *et al.*, 2011 ▸; Leonova *et al.*, 2010 ▸; El-Subbagh *et al.*, 2000 ▸; Jerom & Spencer, 1988 ▸). This wide range of biological activities prompted us to synthesize novel 2,6-diphenyl piperdine-4-one derivatives with enhanced biological activities. In a continuation of this work, the title compound was synthesized using a one-pot Mannich condensation reaction as reported by Noller & Baliah (1948 ▸). The adopted one-pot reaction is convenient, simple, easy way for separation of the product with possible high yield. A Hirshfield surface analysis of the title compound was carried out in order to study how different functionalities can affect the crystal packing.
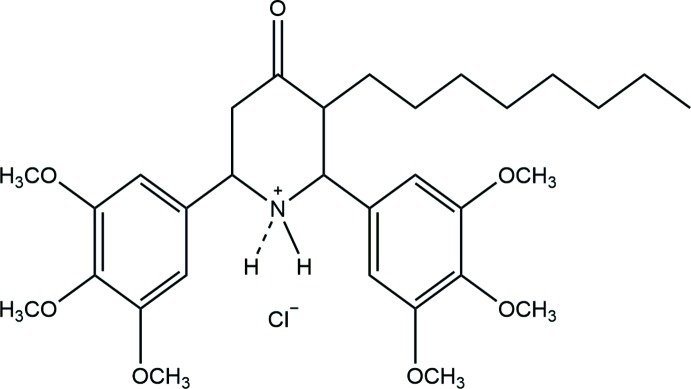


## Structural commentary   

**Figure 1:**
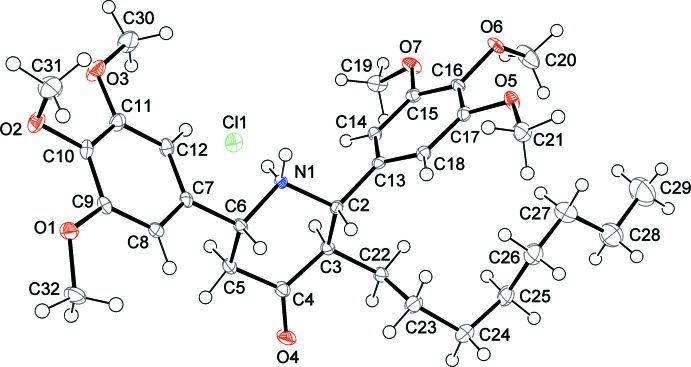
The mol­ecular structure of the title compound with displacement ellipsoids drawn at the 50% probability level.

In the mol­ecule of the title compound (Fig. 1 ▸), the heterocyclic six-membered 4-piperidone ring (N1/C2–C6) adopts a chair conformation, with puckering parameters *Q* = 0.5750 (15) Å, θ = 13.60 (14)° and φ =5.55 (61)°. The octyl chain at C3, and the trimeth­oxy-substituted benzene rings attached at C2 and C6 are equatorially oriented. The trimeth­oxy benzene rings C7–C12 and C13–C18 form a dihedral angle of 73.91 (5)°, and are tilted with respect to the mean plane of the piperidone ring by 59.42 (4) and 78.54 (6)°, respectively. The C13—C2—C3—C22 and O4—C4—C3—C22 torsion angles are 56.36 (17) and −11.0 (2)°, respectively.

## Supra­molecular features   

**Figure 2:**
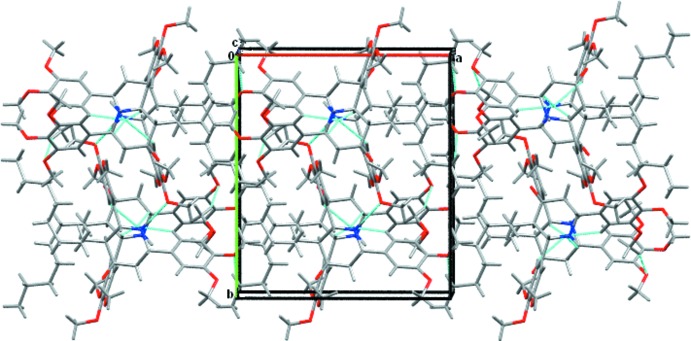
Packing diagram of the title compound viewed approximately along the *c* axis. Turquoise lines indicate hydrogen bonds.

In the crystal, centrosymmetrically-related mol­ecules are linked into dimers through pairs of N—H⋯O hydrogen bonds (Table 1 ▸) forming rings with an 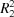
(16) graph-set motif. The dimers are further connected by N—H⋯Cl and C—H⋯Cl hydrogen inter­actions, forming layers parallel to the (011) plane (Fig. 2 ▸).

## Hirshfeld surface analysis   

**Figure 3:**
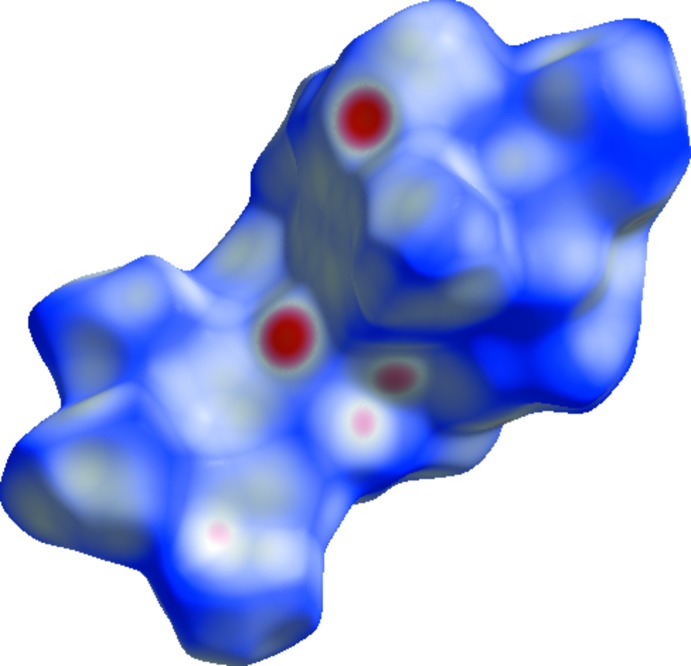
Hirshfeld surface mapped ove *d*_norm_ showing the intermolecular contacts in the title compound.

**Figure 4:**
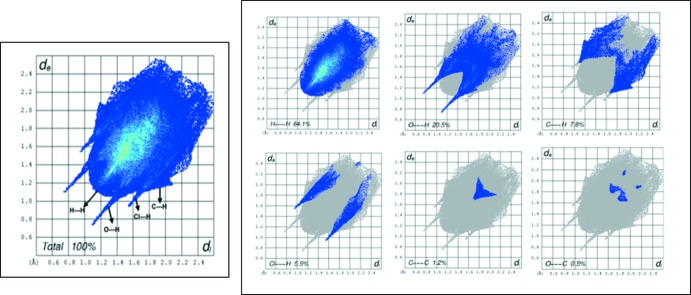
Two-dimensional fingerprint plots for the title compound.

A qu­anti­tative analysis of all type of inter­actions in the title compound was performed using Hirshfeld surface analysis. The Hirshfeld surface mapped over *d*_norm_ (Spackman & Jayatilaka, 2009 ▸) is shown in Fig. 3 ▸ where the red areas on the surface indicate short contacts (as compared to the sum of the van der Waals radii), while the blue areas indicate longer contacts and white areas indicate contacts with distances equal to the sum of the van der Waals radii. Two-dimensional fingerprint plots are shown in Fig. 4 ▸ with a broad hump showing H⋯H contacts and intense spikes indicating a strong O⋯H inter­action, while the broadening in the wing of the C⋯H inter­action is due to the presence of a Cl⋯H inter­action·The largest contribution is from H⋯H inter­actions (64.1%), followed by O⋯H inter­actions, contributing 20.5%. Other weak inter­molecular inter­actions are: C⋯H (7.8%), Cl⋯H (5.5%), C⋯C(1.2%), C⋯O (0.5%) and Cl⋯O (0.4%).

## Database survey   

A search of the Cambridge Crystallographic Database (CSD version 5.39, updates February 2018; Groom *et al.*, 2016 ▸) revealed three examples of organic compounds having piperdine-4-one as the central unit, namely 1-acryloyl-3-methyl-2,6-bis­(3,4,5-tri­meth­oxy­phen­yl)piperidine-4-one (Gnanendra *et al.*, 2009 ▸), *N*-nitroso-2,6-di(3,4,5-tri­meth­oxy­phen­yl)-3,5-di­methyl­piperidin-4-one (Kumaran, *et al.*, 1999 ▸) and 1-(2-chloro­acet­yl)-3-methyl-2,6-bis­(3,4,5-tri­meth­oxy­phen­yl)pip­er­idine-4-one (Lakshminarayana *et al.*, 2009 ▸). A study of the supra­molecular features of these compounds revealed that the crystal lattices are stabilized mainly by C—H⋯O inter­molecular inter­actions, forming two-dimensional networks.

## Synthesis and crystallization   

The title compound was synthesized according to the procedure given in literature (Noller & Baliah, 1948 ▸). A mixture of 2-undeca­none, (0.206 ml, 1 mmol), 3,4,5-tri­meth­oxy­benzaldehyde (0.39 g, 2 mmol) and ammonium acetate (0.077 g, 1 mmol) in ethanol (50 ml) was allowed to reflux for three hours. The progress of reaction was monitored by TLC. After completion of the reaction, the mixture was acidified with dilute hydro­chloric acid (5 mL) and the resulting precipitate was collected, washed with an ethanol–ether mixture (1:4 *v*/*v*), dried and redissolved in ethanol. Crystals suitable for single-crystal X-ray diffraction analysis were obtained on slow evaporation of the solvent at room temperature.

## Refinement   

Crystal data, data collection and structure refinement details are summarized in Table 2 ▸. H atoms on methyl, methyl­ene and benzene were positioned geometrically with C—H = 0.95–1.00 Å and constrained to ride on their parent atoms with *U*_iso_(H) = 1.2*U*_eq_(C) or 1.5*U*_eq_(C) for methyl H atoms. A rotating model was used for the methyl groups. The N-bound hydrogen atoms were located in a difference-Fourier map and freely refined.

## Supplementary Material

Crystal structure: contains datablock(s) global, I. DOI: 10.1107/S2056989018008125/rz5236sup1.cif

Structure factors: contains datablock(s) I. DOI: 10.1107/S2056989018008125/rz5236Isup2.hkl

Supporting information file. DOI: 10.1107/S2056989018008125/rz5236Isup3.cml

CCDC reference: 1846705

Additional supporting information:  crystallographic information; 3D view; checkCIF report

## Figures and Tables

**Table 1:** Hydrogen-bond geometry (Å, °)

*D*—H⋯*A*	*D*—H	H⋯*A*	*D*⋯*A*	*D*—H⋯*A*
N1—H1*A*⋯O6^i^	0.92 (2)	1.93 (2)	2.8500 (18)	175.9 (19)
N1—H2*A*⋯Cl1	0.92 (2)	2.18 (2)	3.0959 (15)	172.6 (19)
C6—H6⋯Cl1^ii^	1.00	2.74	3.6526 (17)	152
C2—H2⋯Cl1^ii^	1.00	2.57	3.5153 (16)	158
C12—H12⋯Cl1	0.95	2.83	3.6625 (18)	147
C14—H14⋯Cl1	0.95	2.82	3.6144 (16)	141
C28—H28*B*⋯O2^iii^	0.99	2.52	3.308 (3)	136

Symmetry codes: (i) 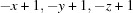; (ii) 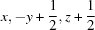; (iii) 
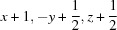
.

**Table 2:** Experimental details

Crystal data	
Chemical formula	C_31_H_46_NO_7_^+^·Cl^−^
*M*_r_	580.14
Crystal system, space group	Monoclinic, *P*2_1_/*c*
Temperature (K)	100
*a*, *b*, *c* (Å)	14.1073 (3), 16.0156 (3), 13.7785 (3)
β (°)	95.006 (1)
*V* (Å^3^)	3101.20 (11)
*Z*	4
Radiation type	Cu *K*α
μ (mm^−1^)	1.47
Crystal size (mm)	0.20 × 0.13 × 0.06

Data collection	
Diffractometer	Bruker APEXII CCD
Absorption correction	Multi-scan (*SADABS*; Bruker, 2014 ▸)
*T*_min_, *T*_max_	0.758, 0.917
No. of measured, independent and observed [*I* > 2σ(*I*)] reflections	42615, 5681, 4654
*R*_int_	0.070
(sin θ/λ)_max_ (Å^−1^)	0.602

Refinement	
*R*[*F* ^2^ > 2σ(*F* ^2^)], *wR*(*F* ^2^), *S*	0.038, 0.094, 1.01
No. of reflections	5681
No. of parameters	376
H-atom treatment	H atoms treated by a mixture of independent and constrained refinement
Δρ_max_, Δρ_min_ (e Å^−3^)	0.30, −0.26

Computer programs: *APEX3* and *SAINT* (Bruker, 2014 ▸), *SHELXT* (Sheldrick, 2015*a* ▸), *SHELXL2016* (Sheldrick, 2015*b* ▸) and *SHELXTL* (Sheldrick, 2008 ▸).

## supplementary crystallographic information

### Crystal data

**Table:**

C_31_H_46_NO_7_^+^·Cl^−^	*F*(000) = 1248
*M**_r_* = 580.14	*D*_x_ = 1.243 Mg m^−^^3^
Monoclinic, *P*2_1_/*c*	Cu *K*α radiation, λ = 1.54178 Å
*a* = 14.1073 (3) Å	Cell parameters from 9910 reflections
*b* = 16.0156 (3) Å	θ = 4.2–68.1°
*c* = 13.7785 (3) Å	µ = 1.47 mm^−^^1^
β = 95.006 (1)°	*T* = 100 K
*V* = 3101.20 (11) Å^3^	Plate, colourless
*Z* = 4	0.20 × 0.13 × 0.06 mm

### Data collection

**Table:**

Bruker APEXII CCD diffractometer	4654 reflections with *I* > 2σ(*I*)
φ and ω scans	*R*_int_ = 0.070
Absorption correction: multi-scan (SADABS; Bruker, 2014)	θ_max_ = 68.2°, θ_min_ = 3.1°
*T*_min_ = 0.758, *T*_max_ = 0.917	*h* = −16→16
42615 measured reflections	*k* = −19→19
5681 independent reflections	*l* = −16→16

### Refinement

**Table:**

Refinement on *F*^2^	0 restraints
Least-squares matrix: full	Hydrogen site location: mixed
*R*[*F*^2^ > 2σ(*F*^2^)] = 0.038	H atoms treated by a mixture of independent and constrained refinement
*wR*(*F*^2^) = 0.094	*w* = 1/[σ^2^(*F*_o_^2^) + (0.0429*P*)^2^ + 1.6956*P*] where *P* = (*F*_o_^2^ + 2*F*_c_^2^)/3
*S* = 1.01	(Δ/σ)_max_ < 0.001
5681 reflections	Δρ_max_ = 0.30 e Å^−^^3^
376 parameters	Δρ_min_ = −0.25 e Å^−^^3^

### Special details

**Table:**

Geometry. All esds (except the esd in the dihedral angle between two l.s. planes) are estimated using the full covariance matrix. The cell esds are taken into account individually in the estimation of esds in distances, angles and torsion angles; correlations between esds in cell parameters are only used when they are defined by crystal symmetry. An approximate (isotropic) treatment of cell esds is used for estimating esds involving l.s. planes.

### Fractional atomic coordinates and isotropic or equivalent isotropic displacement parameters (Å^2^)

**Table:**

	*x*	*y*	*z*	*U*_iso_*/*U*_eq_	
Cl1	0.47703 (3)	0.27339 (3)	0.21759 (3)	0.01915 (11)	
N1	0.45438 (9)	0.26752 (8)	0.43896 (10)	0.0101 (3)	
H1A	0.4132 (15)	0.3058 (13)	0.4627 (15)	0.027 (6)*	
H2A	0.4593 (14)	0.2742 (12)	0.3734 (16)	0.022 (5)*	
O1	0.10007 (8)	0.06252 (7)	0.49865 (9)	0.0175 (3)	
O2	0.02051 (8)	0.16016 (8)	0.35747 (9)	0.0204 (3)	
O3	0.12362 (9)	0.26030 (8)	0.25424 (9)	0.0254 (3)	
O4	0.63342 (9)	0.06866 (7)	0.46982 (10)	0.0244 (3)	
O5	0.62986 (8)	0.55158 (7)	0.64533 (8)	0.0162 (3)	
O6	0.67065 (8)	0.61776 (7)	0.47872 (8)	0.0149 (3)	
O7	0.66126 (9)	0.52733 (7)	0.31193 (8)	0.0185 (3)	
C12	0.27045 (12)	0.22067 (10)	0.34915 (12)	0.0148 (3)	
H12	0.308243	0.255742	0.312448	0.018*	
C11	0.17271 (12)	0.21537 (11)	0.32608 (12)	0.0162 (4)	
C10	0.11760 (11)	0.16272 (11)	0.37882 (12)	0.0147 (3)	
C9	0.16088 (12)	0.11261 (10)	0.45268 (12)	0.0132 (3)	
C8	0.25830 (12)	0.11864 (10)	0.47765 (12)	0.0127 (3)	
H8	0.287831	0.085463	0.528866	0.015*	
C7	0.31232 (11)	0.17396 (10)	0.42668 (11)	0.0117 (3)	
C6	0.41701 (11)	0.18174 (10)	0.45963 (12)	0.0126 (3)	
H6	0.424711	0.173024	0.531757	0.015*	
C5	0.47936 (12)	0.11751 (10)	0.41315 (12)	0.0153 (4)	
H5A	0.458726	0.060623	0.429937	0.018*	
H5B	0.470758	0.123374	0.341395	0.018*	
C4	0.58324 (12)	0.12818 (10)	0.44703 (12)	0.0154 (4)	
C3	0.62160 (11)	0.21731 (10)	0.44544 (12)	0.0130 (3)	
H3	0.623803	0.232932	0.375406	0.016*	
C2	0.55295 (11)	0.27918 (10)	0.48908 (11)	0.0116 (3)	
H2	0.550461	0.264836	0.559444	0.014*	
C13	0.58429 (11)	0.36970 (10)	0.48314 (12)	0.0119 (3)	
C14	0.60889 (11)	0.40364 (10)	0.39611 (11)	0.0126 (3)	
H14	0.605537	0.370723	0.338550	0.015*	
C15	0.63859 (11)	0.48660 (10)	0.39415 (12)	0.0135 (3)	
C16	0.64548 (11)	0.53410 (10)	0.47927 (12)	0.0124 (3)	
C17	0.62075 (11)	0.49956 (10)	0.56647 (11)	0.0124 (3)	
C18	0.58935 (11)	0.41689 (10)	0.56850 (12)	0.0124 (3)	
H18	0.571624	0.393067	0.627422	0.015*	
C19	0.67804 (13)	0.47581 (11)	0.23027 (12)	0.0203 (4)	
H19A	0.618376	0.449091	0.205160	0.031*	
H19B	0.725041	0.432818	0.250694	0.031*	
H19C	0.702184	0.510253	0.179043	0.031*	
C20	0.76737 (14)	0.63474 (12)	0.45957 (17)	0.0325 (5)	
H20A	0.775977	0.621427	0.391473	0.049*	
H20B	0.810579	0.600435	0.502496	0.049*	
H20C	0.781464	0.693938	0.471681	0.049*	
C21	0.60812 (13)	0.51742 (11)	0.73677 (12)	0.0197 (4)	
H21A	0.543104	0.495307	0.730657	0.030*	
H21B	0.613472	0.561252	0.786577	0.030*	
H21C	0.652913	0.472311	0.755689	0.030*	
C22	0.72327 (12)	0.22561 (11)	0.49349 (12)	0.0163 (4)	
H22A	0.763183	0.181857	0.466739	0.020*	
H22B	0.749019	0.280445	0.475552	0.020*	
C23	0.73200 (13)	0.21822 (12)	0.60461 (13)	0.0234 (4)	
H23A	0.702708	0.164977	0.622949	0.028*	
H23B	0.695760	0.264279	0.631877	0.028*	
C24	0.83497 (14)	0.22117 (12)	0.65040 (15)	0.0280 (4)	
H24A	0.870426	0.174045	0.624341	0.034*	
H24B	0.834689	0.212755	0.721557	0.034*	
C25	0.88844 (13)	0.30248 (12)	0.63262 (15)	0.0259 (4)	
H25A	0.955429	0.296223	0.659602	0.031*	
H25B	0.888501	0.311198	0.561497	0.031*	
C26	0.84674 (14)	0.37950 (12)	0.67733 (16)	0.0297 (5)	
H26A	0.856737	0.375278	0.749176	0.036*	
H26B	0.777271	0.380692	0.659265	0.036*	
C27	0.89003 (16)	0.46103 (13)	0.64518 (17)	0.0373 (5)	
H27A	0.877272	0.465999	0.573615	0.045*	
H27B	0.857150	0.507928	0.674904	0.045*	
C28	0.99598 (17)	0.47047 (15)	0.67097 (19)	0.0454 (6)	
H28A	1.029899	0.426339	0.637480	0.055*	
H28B	1.009964	0.462496	0.742032	0.055*	
C29	1.0331 (2)	0.55540 (16)	0.6425 (2)	0.0583 (8)	
H29A	1.016827	0.564801	0.572783	0.087*	
H29B	1.102427	0.557025	0.656495	0.087*	
H29C	1.004092	0.599083	0.679967	0.087*	
C32	0.14052 (13)	−0.00537 (11)	0.55530 (14)	0.0227 (4)	
H32A	0.181150	−0.038465	0.515784	0.034*	
H32B	0.089482	−0.040705	0.576454	0.034*	
H32C	0.178699	0.016486	0.612540	0.034*	
C31	−0.02712 (14)	0.21502 (13)	0.41916 (16)	0.0300 (5)	
H31A	−0.000302	0.271289	0.415775	0.045*	
H31B	−0.018551	0.194756	0.486463	0.045*	
H31C	−0.095136	0.216619	0.397507	0.045*	
C30	0.17655 (14)	0.31979 (13)	0.20387 (14)	0.0280 (5)	
H30A	0.227213	0.291127	0.172634	0.042*	
H30B	0.204707	0.361135	0.250255	0.042*	
H30C	0.134083	0.347957	0.154131	0.042*	

### Atomic displacement parameters (Å^2^)

**Table:**

	*U*^11^	*U*^22^	*U*^33^	*U*^12^	*U*^13^	*U*^23^
Cl1	0.0236 (2)	0.0237 (2)	0.01052 (18)	−0.00088 (17)	0.00375 (15)	−0.00094 (16)
N1	0.0100 (7)	0.0096 (7)	0.0109 (7)	−0.0013 (5)	0.0021 (5)	0.0001 (6)
O1	0.0126 (6)	0.0157 (6)	0.0245 (6)	−0.0022 (5)	0.0034 (5)	0.0052 (5)
O2	0.0103 (6)	0.0270 (7)	0.0234 (6)	−0.0016 (5)	−0.0009 (5)	0.0015 (5)
O3	0.0176 (7)	0.0345 (8)	0.0227 (7)	−0.0059 (6)	−0.0058 (5)	0.0145 (6)
O4	0.0168 (6)	0.0122 (6)	0.0437 (8)	0.0025 (5)	−0.0005 (6)	0.0020 (6)
O5	0.0217 (6)	0.0146 (6)	0.0129 (6)	−0.0038 (5)	0.0037 (5)	−0.0035 (5)
O6	0.0157 (6)	0.0084 (6)	0.0217 (6)	−0.0021 (4)	0.0071 (5)	−0.0009 (5)
O7	0.0306 (7)	0.0125 (6)	0.0134 (6)	−0.0001 (5)	0.0084 (5)	0.0019 (5)
C12	0.0153 (8)	0.0159 (9)	0.0136 (8)	−0.0045 (7)	0.0030 (6)	−0.0009 (7)
C11	0.0174 (9)	0.0193 (9)	0.0114 (8)	−0.0014 (7)	−0.0013 (6)	0.0006 (7)
C10	0.0110 (8)	0.0177 (9)	0.0153 (8)	−0.0033 (7)	0.0003 (6)	−0.0039 (7)
C9	0.0150 (8)	0.0110 (8)	0.0141 (8)	−0.0027 (6)	0.0038 (6)	−0.0033 (6)
C8	0.0148 (8)	0.0103 (8)	0.0131 (8)	0.0002 (6)	0.0019 (6)	−0.0014 (6)
C7	0.0125 (8)	0.0104 (8)	0.0123 (8)	−0.0001 (6)	0.0020 (6)	−0.0049 (6)
C6	0.0143 (8)	0.0103 (8)	0.0132 (8)	−0.0025 (6)	0.0018 (6)	0.0011 (6)
C5	0.0159 (9)	0.0111 (8)	0.0186 (8)	−0.0012 (7)	0.0006 (7)	−0.0022 (7)
C4	0.0144 (9)	0.0132 (9)	0.0187 (8)	0.0001 (7)	0.0022 (7)	−0.0016 (7)
C3	0.0119 (8)	0.0116 (8)	0.0154 (8)	−0.0005 (6)	0.0010 (6)	0.0008 (6)
C2	0.0108 (8)	0.0126 (8)	0.0112 (7)	−0.0016 (6)	−0.0011 (6)	0.0013 (6)
C13	0.0075 (8)	0.0119 (8)	0.0160 (8)	0.0013 (6)	−0.0007 (6)	0.0019 (7)
C14	0.0139 (8)	0.0118 (8)	0.0121 (8)	0.0008 (6)	0.0006 (6)	−0.0020 (6)
C15	0.0126 (8)	0.0141 (8)	0.0139 (8)	0.0008 (6)	0.0019 (6)	0.0023 (7)
C16	0.0101 (8)	0.0086 (8)	0.0185 (8)	−0.0004 (6)	0.0015 (6)	0.0004 (6)
C17	0.0097 (8)	0.0139 (8)	0.0134 (8)	0.0012 (6)	0.0005 (6)	−0.0022 (7)
C18	0.0104 (8)	0.0145 (8)	0.0124 (8)	−0.0005 (6)	0.0013 (6)	0.0010 (6)
C19	0.0282 (10)	0.0210 (9)	0.0129 (8)	0.0044 (8)	0.0076 (7)	0.0021 (7)
C20	0.0233 (11)	0.0205 (10)	0.0565 (14)	−0.0085 (8)	0.0189 (10)	−0.0066 (10)
C21	0.0267 (10)	0.0194 (9)	0.0133 (8)	−0.0008 (7)	0.0027 (7)	−0.0015 (7)
C22	0.0115 (8)	0.0132 (8)	0.0239 (9)	−0.0013 (7)	0.0002 (7)	0.0014 (7)
C23	0.0207 (10)	0.0235 (10)	0.0248 (9)	−0.0045 (8)	−0.0049 (8)	0.0077 (8)
C24	0.0238 (10)	0.0252 (10)	0.0327 (11)	0.0006 (8)	−0.0094 (8)	0.0039 (9)
C25	0.0148 (9)	0.0276 (11)	0.0337 (11)	0.0007 (8)	−0.0067 (8)	−0.0037 (9)
C26	0.0226 (10)	0.0281 (11)	0.0372 (11)	0.0016 (8)	−0.0052 (9)	−0.0043 (9)
C27	0.0430 (13)	0.0247 (11)	0.0427 (13)	0.0052 (9)	−0.0047 (10)	−0.0040 (10)
C28	0.0458 (14)	0.0326 (13)	0.0557 (15)	−0.0129 (11)	−0.0076 (12)	0.0090 (11)
C29	0.073 (2)	0.0385 (15)	0.0618 (17)	−0.0215 (13)	−0.0015 (15)	0.0061 (13)
C32	0.0198 (10)	0.0145 (9)	0.0342 (10)	−0.0017 (7)	0.0043 (8)	0.0080 (8)
C31	0.0163 (10)	0.0279 (11)	0.0463 (12)	0.0036 (8)	0.0056 (9)	0.0000 (9)
C30	0.0284 (11)	0.0326 (11)	0.0221 (9)	−0.0065 (9)	−0.0039 (8)	0.0132 (9)

### Geometric parameters (Å, º)

**Table:**

N1—C6	1.507 (2)	C18—H18	0.9500
N1—C2	1.5092 (19)	C19—H19A	0.9800
N1—H1A	0.92 (2)	C19—H19B	0.9800
N1—H2A	0.92 (2)	C19—H19C	0.9800
O1—C9	1.369 (2)	C20—H20A	0.9800
O1—C32	1.428 (2)	C20—H20B	0.9800
O2—C10	1.3762 (19)	C20—H20C	0.9800
O2—C31	1.430 (2)	C21—H21A	0.9800
O3—C11	1.363 (2)	C21—H21B	0.9800
O3—C30	1.427 (2)	C21—H21C	0.9800
O4—C4	1.212 (2)	C22—C23	1.530 (2)
O5—C17	1.3664 (19)	C22—H22A	0.9900
O5—C21	1.431 (2)	C22—H22B	0.9900
O6—C16	1.3863 (19)	C23—C24	1.533 (2)
O6—C20	1.438 (2)	C23—H23A	0.9900
O7—C15	1.3691 (19)	C23—H23B	0.9900
O7—C19	1.431 (2)	C24—C25	1.535 (3)
C12—C11	1.390 (2)	C24—H24A	0.9900
C12—C7	1.393 (2)	C24—H24B	0.9900
C12—H12	0.9500	C25—C26	1.520 (3)
C11—C10	1.394 (2)	C25—H25A	0.9900
C10—C9	1.394 (2)	C25—H25B	0.9900
C9—C8	1.391 (2)	C26—C27	1.524 (3)
C8—C7	1.397 (2)	C26—H26A	0.9900
C8—H8	0.9500	C26—H26B	0.9900
C7—C6	1.512 (2)	C27—C28	1.513 (3)
C6—C5	1.530 (2)	C27—H27A	0.9900
C6—H6	1.0000	C27—H27B	0.9900
C5—C4	1.508 (2)	C28—C29	1.521 (3)
C5—H5A	0.9900	C28—H28A	0.9900
C5—H5B	0.9900	C28—H28B	0.9900
C4—C3	1.527 (2)	C29—H29A	0.9800
C3—C22	1.532 (2)	C29—H29B	0.9800
C3—C2	1.544 (2)	C29—H29C	0.9800
C3—H3	1.0000	C32—H32A	0.9800
C2—C13	1.520 (2)	C32—H32B	0.9800
C2—H2	1.0000	C32—H32C	0.9800
C13—C14	1.388 (2)	C31—H31A	0.9800
C13—C18	1.395 (2)	C31—H31B	0.9800
C14—C15	1.394 (2)	C31—H31C	0.9800
C14—H14	0.9500	C30—H30A	0.9800
C15—C16	1.394 (2)	C30—H30B	0.9800
C16—C17	1.395 (2)	C30—H30C	0.9800
C17—C18	1.397 (2)		
			
C6—N1—C2	110.48 (12)	H19B—C19—H19C	109.5
C6—N1—H1A	107.3 (13)	O6—C20—H20A	109.5
C2—N1—H1A	109.8 (13)	O6—C20—H20B	109.5
C6—N1—H2A	110.5 (12)	H20A—C20—H20B	109.5
C2—N1—H2A	106.8 (12)	O6—C20—H20C	109.5
H1A—N1—H2A	111.9 (17)	H20A—C20—H20C	109.5
C9—O1—C32	117.53 (13)	H20B—C20—H20C	109.5
C10—O2—C31	111.41 (13)	O5—C21—H21A	109.5
C11—O3—C30	116.83 (13)	O5—C21—H21B	109.5
C17—O5—C21	117.03 (13)	H21A—C21—H21B	109.5
C16—O6—C20	115.55 (13)	O5—C21—H21C	109.5
C15—O7—C19	116.26 (13)	H21A—C21—H21C	109.5
C11—C12—C7	119.23 (15)	H21B—C21—H21C	109.5
C11—C12—H12	120.4	C23—C22—C3	114.61 (14)
C7—C12—H12	120.4	C23—C22—H22A	108.6
O3—C11—C12	124.38 (15)	C3—C22—H22A	108.6
O3—C11—C10	115.27 (15)	C23—C22—H22B	108.6
C12—C11—C10	120.33 (15)	C3—C22—H22B	108.6
O2—C10—C11	119.78 (15)	H22A—C22—H22B	107.6
O2—C10—C9	120.21 (15)	C22—C23—C24	113.59 (16)
C11—C10—C9	120.01 (15)	C22—C23—H23A	108.8
O1—C9—C8	124.86 (15)	C24—C23—H23A	108.8
O1—C9—C10	115.01 (14)	C22—C23—H23B	108.8
C8—C9—C10	120.07 (15)	C24—C23—H23B	108.8
C9—C8—C7	119.35 (15)	H23A—C23—H23B	107.7
C9—C8—H8	120.3	C23—C24—C25	114.93 (15)
C7—C8—H8	120.3	C23—C24—H24A	108.5
C12—C7—C8	120.85 (15)	C25—C24—H24A	108.5
C12—C7—C6	121.59 (14)	C23—C24—H24B	108.5
C8—C7—C6	117.56 (14)	C25—C24—H24B	108.5
N1—C6—C7	111.53 (13)	H24A—C24—H24B	107.5
N1—C6—C5	108.08 (13)	C26—C25—C24	114.25 (17)
C7—C6—C5	113.65 (13)	C26—C25—H25A	108.7
N1—C6—H6	107.8	C24—C25—H25A	108.7
C7—C6—H6	107.8	C26—C25—H25B	108.7
C5—C6—H6	107.8	C24—C25—H25B	108.7
C4—C5—C6	111.89 (13)	H25A—C25—H25B	107.6
C4—C5—H5A	109.2	C25—C26—C27	113.49 (18)
C6—C5—H5A	109.2	C25—C26—H26A	108.9
C4—C5—H5B	109.2	C27—C26—H26A	108.9
C6—C5—H5B	109.2	C25—C26—H26B	108.9
H5A—C5—H5B	107.9	C27—C26—H26B	108.9
O4—C4—C5	121.38 (15)	H26A—C26—H26B	107.7
O4—C4—C3	122.61 (15)	C28—C27—C26	115.47 (18)
C5—C4—C3	115.93 (14)	C28—C27—H27A	108.4
C4—C3—C22	113.23 (13)	C26—C27—H27A	108.4
C4—C3—C2	111.05 (13)	C28—C27—H27B	108.4
C22—C3—C2	111.81 (13)	C26—C27—H27B	108.4
C4—C3—H3	106.8	H27A—C27—H27B	107.5
C22—C3—H3	106.8	C27—C28—C29	112.6 (2)
C2—C3—H3	106.8	C27—C28—H28A	109.1
N1—C2—C13	110.75 (12)	C29—C28—H28A	109.1
N1—C2—C3	109.06 (12)	C27—C28—H28B	109.1
C13—C2—C3	113.33 (13)	C29—C28—H28B	109.1
N1—C2—H2	107.8	H28A—C28—H28B	107.8
C13—C2—H2	107.8	C28—C29—H29A	109.5
C3—C2—H2	107.8	C28—C29—H29B	109.5
C14—C13—C18	121.21 (15)	H29A—C29—H29B	109.5
C14—C13—C2	121.05 (14)	C28—C29—H29C	109.5
C18—C13—C2	117.72 (14)	H29A—C29—H29C	109.5
C13—C14—C15	119.22 (15)	H29B—C29—H29C	109.5
C13—C14—H14	120.4	O1—C32—H32A	109.5
C15—C14—H14	120.4	O1—C32—H32B	109.5
O7—C15—C16	115.59 (14)	H32A—C32—H32B	109.5
O7—C15—C14	124.21 (14)	O1—C32—H32C	109.5
C16—C15—C14	120.19 (15)	H32A—C32—H32C	109.5
O6—C16—C15	121.46 (14)	H32B—C32—H32C	109.5
O6—C16—C17	118.14 (14)	O2—C31—H31A	109.5
C15—C16—C17	120.27 (15)	O2—C31—H31B	109.5
O5—C17—C16	115.47 (14)	H31A—C31—H31B	109.5
O5—C17—C18	124.74 (15)	O2—C31—H31C	109.5
C16—C17—C18	119.79 (15)	H31A—C31—H31C	109.5
C13—C18—C17	119.32 (15)	H31B—C31—H31C	109.5
C13—C18—H18	120.3	O3—C30—H30A	109.5
C17—C18—H18	120.3	O3—C30—H30B	109.5
O7—C19—H19A	109.5	H30A—C30—H30B	109.5
O7—C19—H19B	109.5	O3—C30—H30C	109.5
H19A—C19—H19B	109.5	H30A—C30—H30C	109.5
O7—C19—H19C	109.5	H30B—C30—H30C	109.5
H19A—C19—H19C	109.5		
			
C30—O3—C11—C12	−3.7 (3)	C4—C3—C2—N1	−52.26 (17)
C30—O3—C11—C10	175.14 (16)	C22—C3—C2—N1	−179.79 (13)
C7—C12—C11—O3	177.55 (16)	C4—C3—C2—C13	−176.12 (13)
C7—C12—C11—C10	−1.2 (2)	C22—C3—C2—C13	56.36 (17)
C31—O2—C10—C11	−94.91 (19)	N1—C2—C13—C14	−72.60 (18)
C31—O2—C10—C9	85.34 (19)	C3—C2—C13—C14	50.34 (19)
O3—C11—C10—O2	−1.1 (2)	N1—C2—C13—C18	108.71 (15)
C12—C11—C10—O2	177.78 (15)	C3—C2—C13—C18	−128.36 (15)
O3—C11—C10—C9	178.68 (15)	C18—C13—C14—C15	−0.4 (2)
C12—C11—C10—C9	−2.5 (3)	C2—C13—C14—C15	−179.07 (14)
C32—O1—C9—C8	−19.5 (2)	C19—O7—C15—C16	164.77 (14)
C32—O1—C9—C10	163.47 (15)	C19—O7—C15—C14	−16.2 (2)
O2—C10—C9—O1	0.7 (2)	C13—C14—C15—O7	−177.57 (15)
C11—C10—C9—O1	−179.09 (14)	C13—C14—C15—C16	1.5 (2)
O2—C10—C9—C8	−176.50 (14)	C20—O6—C16—C15	−68.8 (2)
C11—C10—C9—C8	3.7 (2)	C20—O6—C16—C17	115.49 (17)
O1—C9—C8—C7	−178.21 (15)	O7—C15—C16—O6	2.1 (2)
C10—C9—C8—C7	−1.3 (2)	C14—C15—C16—O6	−177.05 (14)
C11—C12—C7—C8	3.6 (2)	O7—C15—C16—C17	177.72 (14)
C11—C12—C7—C6	−175.63 (15)	C14—C15—C16—C17	−1.4 (2)
C9—C8—C7—C12	−2.4 (2)	C21—O5—C17—C16	−177.99 (14)
C9—C8—C7—C6	176.93 (14)	C21—O5—C17—C18	1.9 (2)
C2—N1—C6—C7	169.23 (13)	O6—C16—C17—O5	−4.0 (2)
C2—N1—C6—C5	−65.15 (16)	C15—C16—C17—O5	−179.79 (14)
C12—C7—C6—N1	28.7 (2)	O6—C16—C17—C18	176.06 (14)
C8—C7—C6—N1	−150.55 (14)	C15—C16—C17—C18	0.3 (2)
C12—C7—C6—C5	−93.73 (18)	C14—C13—C18—C17	−0.7 (2)
C8—C7—C6—C5	86.98 (18)	C2—C13—C18—C17	178.00 (14)
N1—C6—C5—C4	54.93 (17)	O5—C17—C18—C13	−179.16 (15)
C7—C6—C5—C4	179.29 (14)	C16—C17—C18—C13	0.8 (2)
C6—C5—C4—O4	136.04 (17)	C4—C3—C22—C23	−72.37 (19)
C6—C5—C4—C3	−47.10 (19)	C2—C3—C22—C23	53.98 (19)
O4—C4—C3—C22	−11.0 (2)	C3—C22—C23—C24	176.33 (15)
C5—C4—C3—C22	172.14 (14)	C22—C23—C24—C25	61.5 (2)
O4—C4—C3—C2	−137.80 (17)	C23—C24—C25—C26	63.6 (2)
C5—C4—C3—C2	45.39 (19)	C24—C25—C26—C27	−170.44 (16)
C6—N1—C2—C13	−170.33 (13)	C25—C26—C27—C28	−60.8 (3)
C6—N1—C2—C3	64.30 (16)	C26—C27—C28—C29	−176.3 (2)

### Hydrogen-bond geometry (Å, º)

**Table:**

*D*—H···*A*	*D*—H	H···*A*	*D*···*A*	*D*—H···*A*
N1—H1*A*···O6^i^	0.92 (2)	1.93 (2)	2.8500 (18)	175.9 (19)
N1—H2*A*···Cl1	0.92 (2)	2.18 (2)	3.0959 (15)	172.6 (19)
C6—H6···Cl1^ii^	1.00	2.74	3.6526 (17)	152
C2—H2···Cl1^ii^	1.00	2.57	3.5153 (16)	158
C12—H12···Cl1	0.95	2.83	3.6625 (18)	147
C14—H14···Cl1	0.95	2.82	3.6144 (16)	141
C28—H28*B*···O2^iii^	0.99	2.52	3.308 (3)	136

Symmetry codes: (i) −*x*+1, −*y*+1, −*z*+1; (ii) *x*, −*y*+1/2, *z*+1/2; (iii) *x*+1, −*y*+1/2, *z*+1/2.
